# Improving Breast Cancer Survival Analysis through Competition-Based Multidimensional Modeling

**DOI:** 10.1371/journal.pcbi.1003047

**Published:** 2013-05-09

**Authors:** Erhan Bilal, Janusz Dutkowski, Justin Guinney, In Sock Jang, Benjamin A. Logsdon, Gaurav Pandey, Benjamin A. Sauerwine, Yishai Shimoni, Hans Kristian Moen Vollan, Brigham H. Mecham, Oscar M. Rueda, Jorg Tost, Christina Curtis, Mariano J. Alvarez, Vessela N. Kristensen, Samuel Aparicio, Anne-Lise Børresen-Dale, Carlos Caldas, Andrea Califano, Stephen H. Friend, Trey Ideker, Eric E. Schadt, Gustavo A. Stolovitzky, Adam A. Margolin

**Affiliations:** 1 IBM TJ Watson Research, Yorktown Heights, New York, United States of America; 2 Departments of Medicine and Bioengineering, University of California San Diego, La Jolla, California, United States of America; 3 Sage Bionetworks, Seattle, Washington, United States of America; 4 Division of Public Health Sciences, Fred Hutchinson Cancer Research Center, Seattle, Washington, United States of America; 5 Department of Genetics and Genomic Sciences, Icahn School of Medicine at Mount Sinai, New York, New York, United States of America; 6 Icahn Institute for Genomics and Multiscale Biology, Icahn School of Medicine at Mount Sinai, New York, New York, United States of America; 7 Columbia Initiative in Systems Biology, Columbia University, New York, New York, United States of America; 8 Center for Computational Biology and Bioinformatics, Columbia University, New York, New York, United States of America; 9 Department of Genetics, Institute for Cancer Research, Oslo University Hospital, Oslo, Norway; 10 The K. G. Jebsen Center for Breast Cancer Research, Institute for Clinical Medicine, Faculty of Medicine, University of Oslo, Oslo, Norway; 11 Cambridge Research Institute, Cancer Research UK, Cambridge, United Kingdom; 12 Department of Oncology, University of Cambridge, Cambridge, United Kingdom; 13 Department of Oncology, Division of Cancer Medicine, Surgery and Transplantation, Oslo University Hospital, Oslo, Norway; 14 Laboratory for Epigenetics and Environment, Centre National de Génotypage, CEA, Institut de Génomique, Evry, France; 15 Department of Preventive Medicine, Keck School of Medicine, University of Southern California, Los Angeles, California, United States of America; 16 Department of Clinical Molecular Biology, Division of Medicine, Akershus University Hospital, Ahus, Norway; 17 Department of Pathology and Laboratory Medicine, University of British Colombia, Vancouver, British Colombia, Canada; 18 Molecular Oncology, British Colombia Cancer Research Center, Vancouver, British Colombia, Canada; 19 Cambridge Experimental Cancer Medicine Centre, Cambridge, United Kingdom; 20 Cambridge Breast Unit, Cambridge University Hospital NHS Foundation Trust and NIHR Cambridge Biomedical Research Centre, Addenbrooke's Hospital, Cambridge, United Kingdom; 21 Department of Biomedical Informatics, Columbia University, New York, New York, United States of America; 22 Department of Biochemistry and Molecular Biophysics, Columbia University, New York, New York, United States of America; 23 Institute for Cancer Genetics, Columbia University, Columbia University, New York, New York, United States of America; 24 Herbert Irving Comprehensive Cancer Center, Columbia University, New York, New York, United States of America; New York University, United States of America

## Abstract

Breast cancer is the most common malignancy in women and is responsible for hundreds of thousands of deaths annually. As with most cancers, it is a heterogeneous disease and different breast cancer subtypes are treated differently. Understanding the difference in prognosis for breast cancer based on its molecular and phenotypic features is one avenue for improving treatment by matching the proper treatment with molecular subtypes of the disease. In this work, we employed a competition-based approach to modeling breast cancer prognosis using large datasets containing genomic and clinical information and an online real-time leaderboard program used to speed feedback to the modeling team and to encourage each modeler to work towards achieving a higher ranked submission. We find that machine learning methods combined with molecular features selected based on expert prior knowledge can improve survival predictions compared to current best-in-class methodologies and that ensemble models trained across multiple user submissions systematically outperform individual models within the ensemble. We also find that model scores are highly consistent across multiple independent evaluations. This study serves as the pilot phase of a much larger competition open to the whole research community, with the goal of understanding general strategies for model optimization using clinical and molecular profiling data and providing an objective, transparent system for assessing prognostic models.

## Author Summary

We developed an extensible software framework for sharing molecular prognostic models of breast cancer survival in a transparent collaborative environment and subjecting each model to automated evaluation using objective metrics. The computational framework presented in this study, our detailed post-hoc analysis of hundreds of modeling approaches, and the use of a novel cutting-edge data resource together represents one of the largest-scale systematic studies to date assessing the factors influencing accuracy of molecular-based prognostic models in breast cancer. Our results demonstrate the ability to infer prognostic models with accuracy on par or greater than previously reported studies, with significant performance improvements by using state-of-the-art machine learning approaches trained on clinical covariates. Our results also demonstrate the difficultly in incorporating molecular data to achieve substantial performance improvements over clinical covariates alone. However, improvement was achieved by combining clinical feature data with intelligent selection of important molecular features based on domain-specific prior knowledge. We observe that ensemble models aggregating the information across many diverse models achieve among the highest scores of all models and systematically out-perform individual models within the ensemble, suggesting a general strategy for leveraging the wisdom of crowds to develop robust predictive models.

## Introduction

Breast cancer remains the most common malignancy in females, with more than 200,000 cases of invasive breast cancer diagnosed in the United States annually [1]. Molecular profiling research in the last decade has revealed breast cancer to be a heterogeneous disease [2]–[4], motivating the development of molecular classifiers of breast cancer sub-types to influence diagnosis, prognosis, and treatment.

In 2002, a research study reported a molecular predictor of breast cancer survival [5] based on analysis of gene expression profiles from 295 breast cancer patients with 5 year clinical follow-up. Based on these results, two independent companies developed the commercially available MammaPrint [6] and Oncotype DX [7] assays, which have both been promising in augmenting risk prediction compared to models based only on clinical data. However, their role in clinical decision-making is still being debated.

Based on the success of these initial molecular profiles, a large number of additional signatures have been proposed to identify markers of breast cancer tumor biology that may affect clinical outcome [8]–[13]. Meta-analyses indicate that many of them perform very similarly in terms of risk prediction, and can often be correlated with markers of cell proliferation [14], a well-known predictor of patient outcome [15], especially for ER+ tumors [16], [17]. Therefore, it is much more challenging to identify signatures that provide additional independent and more specific risk prediction performance once accounting for proliferation and clinical factors. Recent studies have even suggested that most random subsets of genes are significantly associated with breast cancer survival, and that the majority (60%) of 48 published signatures did not perform significantly better than models built from the random subsets of genes [18]. Correcting for the confounding effect of proliferation based on an expression marker of cell proliferation removes most of the signal from the 48 published signatures [18].

The difficulties in reaching community consensus regarding the best breast cancer prognosis signatures illustrates a more intrinsic problem whereby researchers are responsible for both developing a model and comparing its performance against alternatives [19]. This phenomenon has been deemed the “self-assessment trap”, referring to the tendency of researchers to unintentionally or intentionally report results favorable to their model. Such self-assessment bias may arise, for example, by choosing assessment statistics for which their model is likely to perform well, selective reporting of performance in the modeling niche where their method is superior, or increased care or expertise in optimizing performance of their method compared to others.

In this work, we explore the use of a research strategy of collaborative competitions as a way to overcome the self-assessment trap. In particular, the competitive component formally separates model development from model evaluation and provides a transparent and objective mechanism for ranking models. The collaborative component allows models to evolve and improve through knowledge sharing, and thereby emphasizes correct and insightful science as the primary objective of the study.

The concept of collaborative competitions is not without precedent and is most evident in crowd-sourcing efforts for harnessing the competitive instincts of a community. Netflix [20] and X-Prize [21] were two early successes in online hosting of data challenges. Commercial initiatives such as Kaggle [22] and Innocentive [23] have hosted many successful online modeling competitions in astronomy, insurance, medicine, and other data-rich disciplines. The MAQC-II project [24] employed blinded evaluations and standardized datasets in the context of a large consortium-based research study to assess modeling factors related to prediction accuracy across 13 different phenotypic endpoints. Efforts such as CASP [25], DREAM [26], and CAFA [27] have created communities around key scientific challenges in structural biology, systems biology, and protein function prediction, respectively. In all cases it has been observed that the best crowd-sourced models usually outperform state-of-the-art off-the-shelf methods.

Despite their success in achieving models with improved performance, existing resources do not provide a general solution for hosting open-access crowd-sourced collaborative competitions due to two primary factors. First, most systems provide participants with a training dataset and require them to submit a vector of predictions for evaluation in the held-out dataset [20], [22], [24], [26], often requiring (only) the winning team to submit a description of their method and sometimes source code to verify reproducibility. While this achieves the goal of objectively assessing models, we believe it fails to achieve an equally important goal of developing a transparent community resource where participants work openly to collaboratively share and evolve models. We overcome this problem by developing a system where participants submit models as re-runnable source code by implementing a simple programmatic API consisting of a train and predict method. Second, some existing systems are designed primarily to leverage crowd-sourcing to develop models for a commercial partner [22], [23] who pays to run the competition and provides a prize to the developer of the best-performing model. Although we support this approach as a creative and powerful method for advancing commercial applications, such a system imposes limitations on the ability of participants to share models openly as well as intellectual property restrictions on the use of models. We overcome this problem by making all models available to the community through an open source license.

In this study, we formed a research group consisting of scientists from 5 institutions across the United States and conducted a collaborative competition to assess the accuracy of prognostic models of breast cancer survival. This research group, called the Federation, was set up as a mechanism for advancing collaborative research projects designed to demonstrate the benefit of team-oriented science. The rest of our group consisted of the organizers of the DREAM project, the Oslo team from the Norwegian Breast Cancer study, and leaders of the Molecular Taxonomy of Breast Cancer International Consortium (METABRIC), who provided a novel dataset consisting of nearly 2,000 breast cancer samples with median 10-year follow-up, detailed clinical information, and genome-wide gene expression and copy number profiling data. In order to create an independent dataset for assessing model consistency, the Oslo team generated novel copy number data on an additional 102 samples (the MicMa cohort), which was combined with gene expression and clinical data for the same samples that was previously put in the public domain by the same research group [4], [28].

The initial study using the METABRIC data focused on unsupervised molecular sub-class discovery [29]. Although some of the reported sub-classes do correlate with survival, the goal of this initial work was not to build prognostic models. Indeed, the models developed in the current study provide more accurate survival predictions than those trained using molecular sub-classes reported in the original work. Therefore, the current study represents the first large-scale attempt to assess prognostic models based on a dataset of this scale and quality of clinical information.

The contributions of this work are two-fold. First, we conducted a detailed post-hoc analysis of all submitted models to determine model characteristics related to prognostic accuracy. Second, we report the development of a novel computational system for hosting community-based collaborative competitions, providing a generalizable framework for participants to build and evaluate transparent, re-runnable, and extensible models. Further, we suggest elements of study design, dataset characteristics, and evaluation criteria used to assess whether the results of a competition-style research study improve on standard approaches. We stress that the transparency enabled by making source code available and providing objective pre-defined scoring criteria allow researchers in future studies to verify reproducibility, improve on our findings, and assess their generalizability in future applications. Thus the results and computational system developed in this work serve as a pilot study for an open community-based competition on prognostic models of breast cancer survival. More generally, we believe this study will serve as the basis for additional competition-based research projects in the future, with the goal of promoting increased transparency and objectivity in genomics research (and other applications) and providing an open framework to collaboratively evolve complex models leading to patient benefit, beyond the sum of the individual efforts, by leveraging the wisdom of crowds.

## Results

### Competition dataset characteristics

We used the METABRIC dataset as the basis of evaluating prognostic models in this study. This dataset contains a total of nearly 2,000 breast cancer samples. 980 of these samples (excluding those with missing survival information) were available for the duration of the collaborative competition phase of this study. An additional 988 samples became available after we had concluded our evaluation in the initial dataset and, fortunately, served as a large additional dataset for assessing the consistency of our findings. For each sample, the dataset contains median 10 year follow-up, 16 clinical covariates (Table 1), and genome-wide gene expression and copy number profiling data, normalized as described in [29], resulting in 48,803 gene expression features and 31,685 copy number features summarized at the gene level (see Methods).

**Table 1:** Association of clinical features with overall survival (OS).

Variable name	spearman(SP)	SP p-val	univariate HR	uni p-val	multivariate HR	multi p-val
ageDiagnosis	−0.05	2.34e-01	1.03	6.71e-07	1.05	3.98e-10
tumorSizeCM	−0.19	1.47e-05	1.16	2.86e-07	1.1	4.74e-03
Lymphnodes (pos vs. neg)	0.24	4.2e-08	1.68	1.84e-04	1.62	5.2e-03
Hormone (treatment vs. no treatment)	−0.09	3.96e-02	1	9.84e-01	0.63	8.72e-03
Radiation (treatment vs. no treatment)	−0.04	3.62e-01	0.84	2.08e-01	0.7	2.19e-02
PR (pos vs. neg)	0.23	2.52e-07	0.53	4.21e-06	0.7	3.44e-02
Grade (ordinal)	−0.15	1.02e-03	2.48	5.79e-03	1.98	5.63e-02
Chemo (treated vs not)	−0.27	5.62e-10	1.62	3.06e-03	1.67	7.37e-02
HER2 (pos vs. neg)	−0.1	2.50e-02	1.78	2.79e-03	1.43	1.84e-01
hist Medullary (vs. ILC)	−0.04	3.6e-01	1.12	8.58e-01	0.62	4.77e-01
hist Mixed (vs. ILC)	0.06	1.63e-01	0.59	1.61e-01	0.83	6.36e-01
ER (pos. vs. neg)	0.14	1.23e-03	0.65	7.78e-03	0.8	7.73e-01
tripleNegative	−0.09	4.05e-02	1.32	1.42e-01	0.89	7.75e-01
hist Inf Duct (vs. ILC)	−0.04	3.89e-01	1.07	8.17e-01	0.93	7.99e-01
hist Muc (vs. ILC)	0	9.38e-01	0.92	8.75e-01	0.9	8.53e-01
ERPR	0.14	1.18e-03	0.65	8.26e-03	1.13	8.78e-01

The columns are: variable name; Spearman correlation between variable and OS for uncensored patients; p-value of the Spearman correlation; Cox proportional hazard ratio (HR) for all patients between individual clinical features and OS; p-values of the HR (Wald test); HR for all patients using all clinical variables and OS; p-value (Wald test) for the HR using all clinical features. The clinical covariates in this table include age at diagnosis (continuous), tumor size in centimeters (continuous), histological grade (ordinal) and whether the patient received hormone therapy (treated vs. untreated), radiotherapy (treated vs. untreated), or chemotherapy (treated vs. untreated). In addition, there are clinical covariates for common breast cancer subtype markers including HER2 status (positive vs. negative), ER status (positive vs. negative) and PR status (positive vs. negative) individually, as well as joint ER and PR status (ERPR) and triple negative status (tripleNegative) when a patient is negative for ER, PR, and HER. Histological types are: medullary carcinomas, mixed invasive, infiltrating ductal carcinomas (IDC), mucinous carcinomas, and infiltrating lobular carcinomas. Histology is treated as a categorical level variable, with ILC as the baseline. The multivariate Cox model also includes site as a categorical level variable to adjust for inclusion site (not reported). In the multivariate analyses ER status/endocrine treatment and chemotherapy/node status will be confounded. The table is sorted on the p-values of the multivariate analysis.

**Figure 1:**
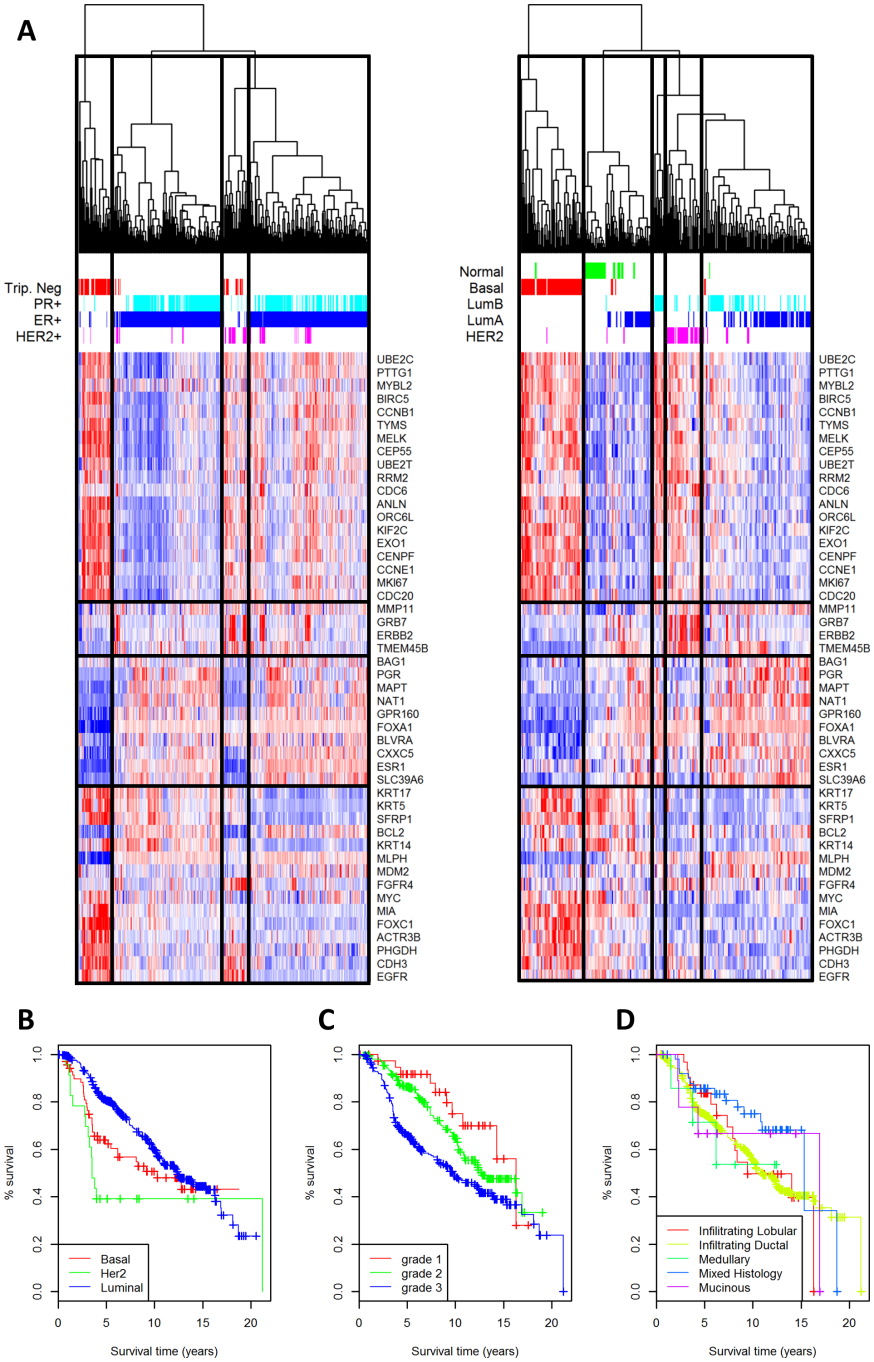
Gene expression subclass analysis. (A) Comparison of hierarchical clustering of METABRIC data (left panel) and Perou data (right panel). Hierarchical clustering on the gene expression data of the PAM50 genes in both datasets reveals a similar gene expression pattern that separates into several subclasses. Although several classes are apparent, they are consistent with sample assignment into basal-like, Her2-enriched and luminal subclasses in the Perou data. Similarly, in the METABRIC data the subclasses are consistent with the available clinical data for triple-negative, ER and PR status, and HER2 positive. (B) Kaplan-Meier plot for subclasses. The METABRIC test dataset was separated into 3 major subclasses according to clinical features. The subclasses were determined by the clinical features: triple negative (red); ER or PR positive status (blue); and HER2 positive with ER and PR negative status (green). The survival curve was estimated using a standard Kaplan-Meier curve, and shows the expected differences in overall survival between the subclasses. (C,D) Kaplan-Meier curve by grade and histology. The test dataset was separated by tumor grade (subplot C; grade 1 – red, grade 2 – green, grade 3- blue), or by histology (subplot D; Infilitrating Lobular – red, Infiltrating Ductal – yellow, Medullary –green, Mixed Histology – blue, or Mucinous - purple). The survival curves were estimated using a standard Kaplan-Meier curve, and show the expected differences in overall survival for the clinical features.

Initial analysis was performed to confirm that the data employed in the competition were consistent with previously published datasets and to identify potential confounding factors such as internal subclasses. Data-driven, unsupervised hierarchical clustering of gene expression levels revealed the heterogeneity of the data and suggested that multiple subclasses do exist (not shown) [29]. However, for the current analysis we decided to focus on the well established separation into basal, luminal, and HER2 positive subclasses, as previously defined [2], [30]. These subclasses are known to closely match clinical data in the following way: most triple-negative samples belong to the basal subclass; most ER positive samples belong to the luminal subclass; and most ER negative HER2 positive samples belong to the HER2 subclass. To ensure that this holds in the current dataset, the 50 genes that best separate the molecular subclasses in the Perou dataset [31] (PAM50) were used for hierarchical clustering of the METABRIC data and compared with a similar clustering of the Perou dataset (Figure 1A). The results of the supervised clustering reveal similar subclasses with similar gene expression signatures as those presented by Perou et al, and were also consistent with the clinical definitions as presented above. Finally, the 3 subclasses show a distinct separation in their Kaplan-Meier overall survival plots for the three subtypes defined by the clinical data, where the HER2 subclass has the worst prognosis, followed by the basal subclass, and the luminal subclass has the best prognosis, as expected (Figure 1B). This analysis shows that sub-classification based on ER (IHC), PR (gene expression), and HER2 (copy number) should capture the major confounding factors that may be introduced by the heterogeneity of the disease.

Multiple individual clinical features exhibit high correlation with survival for non-censored patients, and have well documented prognostic power (Table 1, Figure 1C), while others have little prognostic power (Figure 1D). To demonstrate that the competition data is consistent in this respect, a Cox proportional hazard model was fit to the overall survival (OS) of all patients using each one of the clinical covariates individually. As expected, the most predictive single clinical features are the tumor size, age at diagnosis, PR status, and presence of lymph node metastases (Table 1). To assess the redundancy of the clinical variables, an additional multivariable Cox proportional hazard model was fit to the overall survival (OS) of all patients using all clinical features. The remaining statistically significant covariates were patient age at diagnosis (the most predictive feature), followed by tumor size, presence of lymph node metastases, and whether the patient received hormone therapy.

### Improving breast cancer models in the pilot competition

Participants from our 5 research groups were provided data from 500 patient samples used to train prognostic models. These models were submitted as re-runnable source code and participants were provided real-time feedback in the form of a “leaderboard” based on the concordance index of predicted survival versus the observed survival in the 480 held-out samples.

**Figure 2:**
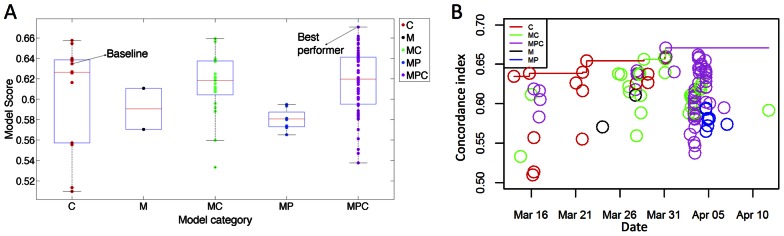
Distribution of concordance index scores of models submitted in the pilot competition. (A) Models are categorized by the type of features they use. Boxes indicate the 25^th^ (lower end), 50^th^ (middle red line) and 75^th^ (upper end) of the scores in each category, while the whiskers indicate the 10^th^ and 90^th^ percentiles of the scores. The scores for the baseline and best performer are highlighted. (B) Model performance by submission date. In the initial phase of the competition, slight improvements over the baseline model were achieved by applying machine learning approaches to only the clinical data (red circles), whereas initial attempts to incorporate molecular data significantly decreased performance (green, purple, and black circles). In the intermediate phase of the competition, models combining molecular and clinical data (green circles) predominated and achieved slightly improved performance over clinical only models. Towards the end of the competition, models combining clinical information with molecular features selected based on prior information (purple circles) predominated.

Participants independently submitted 110 models to predict survival from the supplied clinical and molecular data (Table S1), showing a wide variability in their performance, which was expected since there were no constraints on the submissions. Post-hoc analysis of submitted models revealed 5 broad classes of modeling strategies based on if the model was trained using: only clinical features (C); only molecular features (M); molecular and clinical features (MC); molecular features selected using prior knowledge (MP); molecular features selected using prior knowledge combined with clinical features (MPC) (Table 2). The complete distribution of the performance of all the models, evaluated using concordance index, and classified into these categories is shown in Figure 2.

**Table 2:** Description of categories of models submitted to the pilot competition based on the features used by the models in each category.

Category	Features used by models	# models	Range of c-index (median)
C	Only clinical (C) features	14	0.5097–0.6576 (0.6264)
M	Only molecular (M) features	2	0.5705–0.6108 (0.5906)
MC	Molecular and clinical features	28	0.5334–0.6593 (0.6169)
MP	Molecular features selected using prior (P) knowledge	8	0.5651–0.5947 (0.5806)
MPC	Molecular features selected as above and all clinical features	58	0.5376–0.6707 (0.6197)

Analysis of the relative performance among model categories suggested interesting patterns related to criteria influencing model performance. The traditional method for predicting outcome is Cox regression on the clinical features [32]. This model, which used only clinical features, served as our baseline, and obtained a concordance index of 0.6347 on the validation set. Models trained on the clinical covariates using state-of-the-art machine learning methods (elastic net, lasso, random forest, boosting) achieved notable performance improvements over the baseline Cox regression model (Figure 2, category ‘C’).

Two submitted models were built by naively inputting all molecular features into machine learning algorithms (i.e. using all gene expression and CNA features and no clinical features). These models (our category ‘M’) both performed significantly worse than the baseline clinical model (median concordance index of 0.5906). Given that our training set contains over 80,000 molecular features and only 500 training samples, this result highlights the challenges related to overfitting due to the imbalance between the number of features and number of samples, also known as the curse of dimensionality [33], [34].

Models trained using molecular feature data combined with clinical data (category ‘MC’) outperformed the baseline clinical model in 10 out of 28 (36%) submissions, suggesting there is some difficulty in the naïve incorporation of molecular feature data compared to using only clinical information. In fact, the best MC model attributed lower weights to molecular compared to clinical features by rank-transforming all the features (molecular and clinical) and training an elastic net model, imposing a penalty only on the molecular features and not on the clinical ones, such that the clinical features are always included in the trained model. This model achieved a concordance index of 0.6593, slightly better than the best-performing clinical only model.

One of the most successful approaches to addressing the curse of dimensionality in genomics problems has been to utilize domain-specific prior knowledge to pre-select features more likely to be associated with the phenotype of interest [35]. Indeed, the majority of submitted models (66 of 110, 60%) utilized a strategy of pre-selecting features based on external prior knowledge. Interestingly, analysis of model submission dates indicates that participants first attempted naïve models incorporating all molecular features, and after achieving small performance improvements over clinical only models, evolved to incorporate prior information as the dominant modeling strategy in the later phase of the competition (Figure 2B). This observation is consistent with previous reports highlighting the importance of real-time feedback in motivating participants to build continuously improving models [36].

All models trained on only the molecular features (i.e. excluding the clinical features) and incorporating prior knowledge (MP category) performed worse than the baseline model, with the highest concordance index being 0.5947, further highlighting the difficultly in using molecular information alone to improve prognostic accuracy compared to clinical data.

Twenty-four models outperformed the baseline by combining clinical features with molecular features selected by prior knowledge (MPC category). The overall best-performing model attained a concordance index of 0.6707 by training a machine learning method (boosted regression) on a combination of: 1) clinical features; 2) expression levels of genes selected based on both data driven criteria and prior knowledge of their involvement in breast cancer (the MASP feature selection strategy, as described in Methods); 3) an aggregated “genomic instability” index calculated from the copy number data (see Methods).

The wide range of concordance index scores for models in the MPC category raises the question of whether the improved performance of the best MPC models are explained by the biological relevance of the selected features or simply by random fluctuations in model scores when testing many feature sets. Due to the uncontrolled experimental design inherent in accepting unconstrained model submissions, additional evaluations are needed to assess the impact of different modeling choices in a controlled experimental design. We describe the results of this experiment next.

### Controlled experiment for model evaluation

We analyzed the modeling strategies utilized in the original “uncontrolled” model submission phase and designed a “controlled” experiment to assess the associations of different modeling choices with model performance. We determined that most models developed in the uncontrolled experiment could be described as the combination of a machine learning method with a feature selection strategy. We therefore tested models trained using combinations of a discrete set of machine learning methods crossed with feature selection strategies using the following experimental design:

1. We designed 15 categories of models based on the choice of features used in each model based on the following design:
  - We chose 6 strategies for pre-selecting feature subsets as developed in the uncontrolled phase (Table 3).
  - We created 6 additional model categories consisting of each feature subset plus all clinical covariates.
  - We created an additional model category using only clinical covariates.
  - We created 2 additional categories incorporating the genomic instability index (GII), which was a component of the best-performing model in the uncontrolled phase. We used GII in additional to all clinical covariates, as well as GII in addition to all clinical covariates and the additional features used in the best-performing model (MASP) from the uncontrolled experiment. We note that since GII is only a single feature we did not train models using GII alone.
2. For each of the 15 feature selection strategies described above, we trained 4 separate models using the machine learning algorithms that were frequently applied and demonstrated good performance in the uncontrolled experiment: boosting, random survival forest, lasso, and elastic net.
3. We constructed a series of ensemble learning algorithms by computing concordance index scores after averaging the rank predictions of subsets of models. Models trained using ensemble strategies included:
  - 15 ensemble models combining the learning algorithms for each model category.
  - 4 ensemble models combining the model categories for each learning algorithm.
  - 1 ensemble model combining all model categories and learning algorithms.

**Table 3:** Feature sets used in the controlled experiment.

Feature Category	Description
Clinical	The set of 14 clinical features from [29].
Marginal Association	1000 molecular features (gene expression and/or copy number) most predictive of survival in a univariate Cox regression analysis on the training set.
Top-Varying	1000 molecular features (gene expression and/or copy number) with the greatest variance in the training set.
Cancer Census	1526 gene expression and copy number features corresponding to 487 genes from the Cancer Gene Census database [60].
Higgins	1000 gene expression and copy-number features with the greatest variance among oncogenes identified by Higgins et al. [61].
Metabric Clustering	754 gene expression and copy number features used to define the clusters in the study by Curtis et al. [29].
MASP: Marginal Association with Subsampling and Prior Knowledge	Gene expression of 50 known oncogenes and transcription factors selected by computing univariate Cox regression models on random subsets of the training set and aggregating the resulting p-values (see Methods).
GII: Genomic Instability Index	Number of amplified/deleted sites as calculated from the segmented copy number data (see Methods).

This experiment design resulted in a total of 60 models based on combinations of modeling strategies from the uncontrolled experiment (Table S4), plus 20 models using ensemble strategies. This controlled experimental design allowed us to assess the effect of different modeling choices while holding other factors constant.

Following an approach suggested in the MAQC-II study [24], we designed negative and positive control experiments to infer bounds on model performance in prediction problems for which models should perform poorly and well, respectively. As a negative control, we randomly permuted the sample labels of the survival data, for both the training and test datasets, and computed the concordance index of each model trained and tested on the permuted data. To evaluate how the models would perform on a relatively easy prediction task, we conducted a positive control experiment in which all models were used to predict the ER status of the patients based on selected molecular features (excluding the ER expression measurement). We found that all negative control models scored within a relatively tight range of concordance indices centered around 0.5 (minimum: 0.468, maximum: 0.551), significantly lower than the lowest concordance index (0.575) of any model trained on the real data in this experiment. Conversely, all ER-prediction models scored highly (minimum: 0.79, maximum: 0.969), suggesting that the scores achieved by our survival models (maximum: 0.6707) are not due to a general limitation of the selected modeling strategies but rather the difficulty of modeling breast cancer survival.

**Figure 3:**
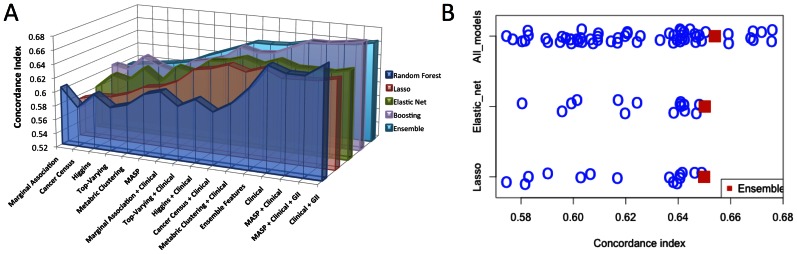
Model performance by feature set and learning algorithm. (A) The concordance index is displayed for each model from the controlled experiment (Table S4). The methods and features sets are arranged according to the mean concordance index score. The ensemble method (cyan curve) infers survival predictions based on the average rank of samples from each of the four other learning algorithms, and the ensemble feature set uses the average rank of samples based on models trained using all of the other feature sets. Results for the METABRIC2 and MicMa datasets are show in Figure S1. (B) The concordance index of models from the controlled phase by type. The ensemble method again utilizes the average rank for models in each category.

Overall, we found that the predictive performance of the controlled experiment models (Figure 3A) was significantly dependent on the individual feature sets (*P* = 1.02e-09, F-test), and less dependent on the choice of the statistical learning algorithm (*P* = 0.23, F-test). All model categories using clinical covariates outperformed all model categories trained excluding clinical covariates, based on the average score across the 4 learning algorithms. The best-performing model category selected features based on marginal correlation with survival, further highlighting the difficulty in purely data-driven approaches, and the need to incorporate prior knowledge to overcome the curse of dimensionality.

The best-performing model used a random survival forest algorithm trained by combining the clinical covariates with a single additional aggregate feature, called the genomic instability index (GII), calculated as the proportion of amplified or deleted sites based on the copy number data. This result highlights the importance of evaluating models using a controlled experimental design, as the best-performing method in the uncontrolled experiment combined clinical variables with GII in addition to selected gene expression features (clinical variables plus only GII was not evaluated), and the controlled experiment pointed to isolating GII as the modeling insight associated with high prediction accuracy.

The random survival forest trained using clinical covariates and GII was significantly better than a random survival forest trained using clinical covariates alone (*P* = 2e-12 by paired Wilcoxon signed rank test based on 100 bootstrap samples with replacement from the test dataset). We also tested if inclusion of the GII feature improved model performance beyond a score that could be obtained by chance based on random selection of features. We trained 100 random survival forest models and 100 boosting models, each utilizing clinical information in addition to random selections of 50 molecular features (corresponding to the number of features used based on the MASP strategy, which achieved the highest score of all feature selection methods). The best-performing model from our competition (trained using clinical covariates and GII) achieved a higher score than each of these 100 models for both learning algorithms (*P*< = .01).

The use of the aggregate GII feature was based on previous reports demonstrating the association between GII and poor prognosis breast cancer subtypes like Luminal B, HER2+ and Basal-like tumors [37]. We found that HER2+ tumors had the strongest association with the GII score (*P* = 1.65e-12, t-test) which partly explains why it performs so well considering none of the patients were treated with compounds that target the HER2 pathway (e.g. Herceptin). Samples with high GII scores were also associated with high-grade tumors (*P* = 7.13e-13, t-test), further strengthening its credential as a good survival predictor. However, despite these strong associations, the genomic instability index provided an added value to the strength of predictions even as clinical covariates histologic grade and HER2 status are used in the models.

Boosting was the best-performing method on average. Elastic net and lasso exhibited stable performance across many feature sets. Random survival forests performed very well when trained on a small number of features based on clinical information and the genomic instability index. However, their performance decreased substantially with the inclusion of large molecular feature sets.

Ensemble methods trained by averaging predicted ranks across multiple methods systematically performed better than the average concordance index scores of the models contained in the ensemble, consistent with previously reported results [38]. Strikingly, an ensemble method aggregating all 60 models achieved a concordance index score of .654, significantly greater than the average of all model scores (.623) (Figure 3B). The ensemble performed better than the average model score for each of 100 resampled collections of 60 models each, using bootstrapping to sample with replacement from all 60 models (*P*< = .01). The ensemble model scored better than 52 of the 60 (87%) models that constituted the ensemble. We note that 2 of the algorithms (boosting and random forests) utilize ensemble learning strategies on their own. For both of the other 2 algorithms (lasso and elastic net) the method trained on an ensemble of the 15 feature sets scored higher than each of the 15 models trained on the individual feature sets (Figure 3B). Consistent with previous reports, the systematic outperformance of ensemble models compared to their constituent parts suggests that ensemble approaches effectively create a consensus that enhances the biologically meaningful signals captured by multiple modeling approaches. As previously suggested in the context of the DREAM project [38]–[41], our finding further reinforces the notion that crowd-sourced collaborative competitions are a powerful framework for developing robust predictive models by training an ensemble model aggregated across diverse strategies employed by participants.

### Consistency of results in independent datasets

In the first round of the competition, we did not restrict the number of models a participant could submit. This raises the possibility of model overfitting to the test set used to provide real-time feedback. We therefore used 2 additional datasets to evaluate the consistency of our findings. The first dataset, which we called METABRIC2, consisted of the 988 samples (excluding those with missing survival data) from the METABRIC cohort that were not used in either the training dataset or the test dataset used for real-time evaluation. The second dataset, called MicMa, consisted of 102 samples with gene expression, clinical covariates, and survival data available [4], [28] and copy number data presented in the current study (see Methods). We used the models from our controlled experiment, which were trained on the original 500 METABRIC samples, and evaluated the concordance index of the survival predictions of each model compared to observed survival in both METABRIC2 and MicMa.

**Figure 4:**
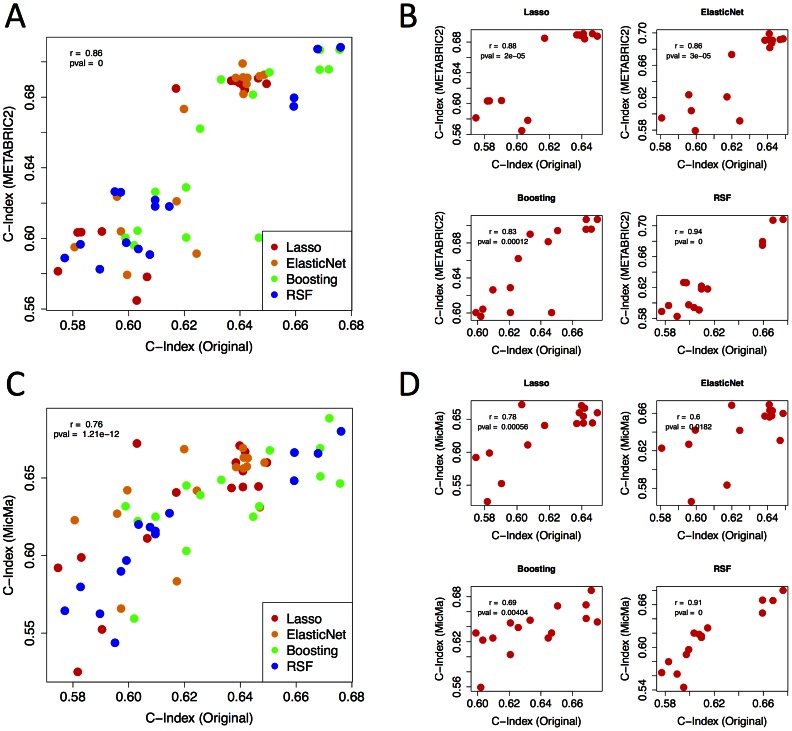
Consistency of results in 2 additional datasets. (A,C) Concordance index scores for all models evaluated in the controlled experiment. Scores from the original evaluation are compared against METABRIC2 (A) and MicMa (C). The 4 machine learning algorithms are displayed in different colors. (B,D) Individual plots for each machine learning algorithm.

The concordance index scores across models from the original evaluation were highly consistent in both METABRIC2 and MicMa. The 60 models evaluated in the controlled experiment (15 feature sets used in 4 learning algorithms) had Pearson correlations of .87 (*P*<1e-10) compared to METABRIC2 (Figure 4A) and .76 (*P*<1e-10) compared to MicMa (Figure 4C), although we note that p-values may be over-estimated due to smaller effective sample sizes due to non-independence of modeling strategies. Model performance was also strongly correlated for each different algorithm across the feature sets for both METABRIC2 (Figure 4B) and MicMa (Figure 4D).

Consistent with results from the original experiment, the top scoring model, based on average concordance index of the METABRIC2 and MicMa scores, was a random survival forest trained using clinical features in combination with the GII. The second best model corresponded to the best model from the uncontrolled experiment (3^rd^ best model in the controlled experiment), and used clinical data in combination with GII and the MASP feature selection strategy, and was trained using a boosting algorithm. A random forest trained using only clinical data achieve the 3^rd^ highest score. The top 39 models all incorporated clinical data.

As an additional comparison, we generated survival predictions based on published procedures used in the clinically approved MammaPrint [6] and Oncotype DX [7] assays. We note that these assays are designed specifically for early stage, invasive, lymph node negative breast cancers (in addition ER+ in the case of Oncotype DX) and use different scores calculated from gene expression data measured on distinct platforms. It is thus difficult to reproduce exactly the predictions provided by these assays or to perform a fair comparison to the present methods on a dataset that includes samples from the whole spectrum of breast tumors. The actual Oncotype DX score is calculated from RT-PCR measurements of the mRNA levels of 21 genes. Using z-score normalized gene expression values from METABRIC2 and MicMa datasets, together with their published weights, we recalculated Oncotype DX scores in an attempt to reproduce the actual scores as closely as possible. We then scored the resulting predictions against the two datasets and obtained concordance indices of 0.6064 for METABRIC2 and 0.5828 for MicMa, corresponding to the 81^st^ ranked model based on average concordance index out of all 97 models tested, including ensemble models and Oncotype DX and MammaPrint feature sets incorporated in all learning algorithms (see Table S5). Similarly, the actual MammaPrint score is calculated based on microarray gene expression measurements, with each patient's score determined by the correlation of the expression of 70 specific genes to the average expression of these genes in patients with good prognosis (defined as those who have no distant metastases for more than five years, ER+ tumors, age less than 55 years old, tumor size less than 5 cm, and are lymph node negative). Because of limitations in the data, we were not able to compute this score in exactly the same manner as the original assay (we did not have the metastases free survival time, and some of the other clinical features were not present in the validation datasets). We estimated the average gene expression profile for the 70 MammaPrint genes based on all patients who lived longer than five years (with standardized gene expression data), then computed each patient's score as their correlation to this average good prognosis profile. We scored the predictions against the two validation datasets and observed concordance indices of 0.602 in METABRIC2 and 0.598 in MicMa, corresponding to the 78^th^ ranked out of 97 models based on average concordance index.

We were able to significantly improve the scores associated with both MammaPrint and Oncotype DX by incorporating the gene expression features utilized by each assay as feature selection criteria in our prediction pipelines. We trained each of the 4 machine learning algorithms with clinical features in addition to gene lists from MammaPrint and Oncotype DX. The best-performing models would have achieved the 8^th^ and 26^th^ best scores, respectively, based on average concordance index in METABRIC2 and MicMa. We note that using the ensemble strategy of combining the 4 algorithms, the model trained using Mammaprint genes and clinical data performed better than clinical data alone, and achieved the 5^th^ highest average model score, including the top score in METABRIC2, slightly (.005 concordance index difference) better than the random forest model using clinical data combined with GII, though only the 17^st^ ranked score in MicMa. This result suggests that incorporating the gene expression features identified by these clinically implemented assays into the prediction pipeline described here may improve prediction accuracy compared to current analysis protocols.

An ensemble method, aggregating results across all learning algorithms and feature sets, performed better than 71 of the 76 models (93%) that constituted the ensemble, consistent with our finding that the ensemble strategy achieves performance among the top individual approaches. For the 19 feature selection strategies used in the METABRIC2 and MicMa evaluations, an ensemble model combining the results of the 4 learning algorithms performed better than the average of the 4 learning algorithms in 36 out of 38 cases (95%). Also consistent with our previous result, for both algorithms that did not use ensemble strategies themselves (elastic net and lasso), an ensemble model aggregating results across the 19 feature sets performed better than each of the individual 19 feature sets for both METABRIC2 and MicMa. Taken together, the independent evaluations in 2 additional datasets are consistent with the conclusions drawn from the original real-time feedback phase of the completion, regarding improvements gained from ensemble strategies and the relative performance of models.

## Discussion

“Precision Medicine”, as defined by the Institute of Medicine Report last year, proposes a world where medical decisions will be guided by molecular markers that ensure therapies are tailored to the patients who receive them [42]. Moving towards this futuristic vision of cancer medicine requires systematic approaches that will help ensure that predictive models of cancer phenotypes are both clinically meaningful and robust to technical and biological sources of variation.

Despite isolated successful developments of molecular diagnostic and personalized medicine applications, such approaches have not translated to routine adoption in standard-of-care protocols. Even in applications where successful molecular tests have been developed, such as breast cancer prognosis [5], [6], a plethora of research studies have claimed to develop models with improved predictive performance. Much of this failure has been attributed to “difficulties in reproducibility, expense, standardization and proof of significance beyond current protocols” [43]. The propensity of researchers to over-report the performance of their own approaches has been deemed the “self-assessment trap” [19].

We propose community-based collaborative competitions [43]–[49] as a general framework to develop and evaluate predictive models of cancer phenotypes from high-throughput molecular profiling data. This approach overcomes limitations associated with the design of typical research studies, which may conflate self-assessment with methodology development or, even more problematic, with data generation. Thus competition-style research may promote transparency and objective assessment of methodologies, promoting the emergence of community standards of methodologies most likely to yield translational clinical benefit.

The primary challenge of any competition framework is to ensure that mechanisms are in place to prevent overfitting and fairly assess model performance, since performance is only meaningful if models are ranked based on their ability to capture some underlying signal in the data. For example, such an approach requires datasets affording sufficient sample sizes and statistical power to make meaningful comparisons of many models across multiple training and testing data subsets. We propose several strategies for assessing if the results obtained from a collaborative competition are likely to generalize to future applications and improve on state-of-the art methodologies that would be employed by an expert analyst.

First, baseline methods should be provided as examples of approaches an experienced analyst may apply to the problem. In our study, we employed a number of such methods for comparison, including methodologies used in clinical diagnostic tests and multiple state-of-the-art machine learning methods trained using only clinical covariates.

Second, performance of models should be evaluated in multiple rounds of independent validation. In this study, we employed a multi-phase strategy suggested by previous researchers [50] in which a portion of the dataset is held back to provide real-time feedback to participants on model performance and another portion of the dataset is held back and used to score the performance of all models, such that participants cannot overfit their models to the test set. If possible, we recommend an additional round of validation using a dataset different from the one used in previous rounds, in order to test against the possibility that good performance is due to modeling confounding variables in the original dataset. This experimental design provides 3 independent rounds of model performance assessment, and consistent results across these multiple evaluations provides strong evidence that performance of the best approaches discovered in this experimental design are likely to generalize in additional datasets.

Finally, statistical permutation tests can provide useful safeguards against the possibility that improved model performance is attributable to random fluctuations based on evaluation of many models. Such tests should be designed carefully based on the appropriate null hypothesis. A useful, though often insufficient, test is to utilize a negative control null model, for example by permuting the sample labels of the response variable. We suggest that additional tests may be employed as post-hoc procedures designed specifically to provide falsifiable hypotheses that may provide alternative explanations of model performance. For example, in this study we assessed the performance of many models trained using the same learning algorithm (random survival forest) and the same clinical features as used in the top scoring model, but using random selections of molecular features instead of the GII feature. This test was designed to falsify the hypothesis that model performance is within the range of likely values based on random selection of features, as has been a criticism of previously reported models [18].

We suggest that the guidelines listed above provide a useful framework in reporting the results of a collaborate competition, and may even be considered necessary criteria to establish the likelihood that findings will generalize to future applications. As with most research studies, a single competition cannot comprehensively assess the full extent to which findings may generalize to all potentially related future applications. Accordingly, we suggest that a collaborative competition should indeed report the best forming model, provided it meets the criteria listed above, but need not focus on declaring a single methodology as conclusively better than all others. By analogy to athletic competitions such as an Olympic track race, a gold medal is given to the runner with the fastest time, even if by a fraction of a second. Judgments of superior athletes emerge through integrating multiple such data points across many races against different opponents, distances, weather conditions, etc., and active debate among the community. A research study framed as a collaborative competition may facilitate the transparency, reproducibility, and objective evaluation criteria that provide the framework on which future studies may build and iterate towards increasingly refined assessments through a continuous community-based effort.

Within several months we developed and evaluated several hundred modeling approaches. Our research group consisted of experienced analysts trained as both data scientists and clinicians, resulting in models representing state-of-the art approaches employed in both machine learning and clinical cancer research (Table 3). By conducting detailed post-hoc analysis of approaches developed by this group, we were able to design a controlled experiment to isolate the performance improvements attributable to different strategies, and to potentially combine aspects of different approaches into a new method with improved performance.

The design of our controlled experiment builds off pioneering work by the MAQC-II consortium, which compiled 6 microarray datasets from the public domain and assessed modeling factors related to the ability to predict 13 different phenotypic endpoints. MAQC-II classified each model based on several factors (type of algorithm, normalization procedure, etc), allowing analysis of the effect of each modeling factor on performance. Our controlled experiment follows this general strategy, and extends it in several ways.

First, MAQC-II, and most competition-base studies [20], [22], [26], accept submissions in the form of prediction vectors. We developed a computational system that accepts models as re-runnable source code implementing a simple train and predict API. Source code for all submitted models are stored in the Synapse compute system [51] and are freely available to the community. Thus researchers may reproduce reported results, verify fair play and lack of cheating, learn from the best-performing models, reuse submitted models in related applications (e.g. building prognostic models in other datasets), build ensemble models by combining results of submitted models, and combine and extend innovative ideas to develop novel approaches. Moreover, storing models as re-runnable source code is important in assessing the generalizability and robustness of models, as we are able to re-train models using different splits or subsets of the data to evaluate robustness, and we (or any researcher) can evaluate generalizability by assessing the accuracy of a model's predictions in an independent dataset, such as existing related studies [5] or emerging clinical trial data [52]. We believe this software system will serve as a general resource that is extended and re-used in many future competition-based studies.

Second, MAQC-II conducted analysis across multiple phenotypic endpoints, which allowed models to be re-evaluated in the context of many prediction problems. However, this design required models to be standardized across all prediction problems and did not allow domain-specific insights to be assessed for each prediction problem. By contrast, our study focused on the single biomedical problem of breast cancer prognosis, and allowed clinical research specialists to incorporate expert knowledge into modeling approaches. In fact, we observed that feature selection strategies based on prior domain-specific knowledge had a greater effect on model performance than the choice of learning algorithm, and learning algorithms that did not incorporate prior knowledge were unable to overcome challenges with incorporating high-dimensional feature data. In contrast to previous reports that have emphasized abstracting away domain-specific aspects of a competition in order to attract a broader set of analysis [50], in real-word problems, we emphasize the benefit of allowing researchers to apply domain-specific expertise and objectively test the performance of such approaches against those of analysts employing a different toolbox of approaches.

Finally, whereas MAQC-II employed training and testing splits of datasets for model evaluation, our study provides an additional level of evaluation in a separate, independent dataset generated on a different cohort and using different gene expression and copy number profiling technology. Consistent with findings reported by MAQC-II, our study demonstrates strong consistency of model performance across independent evaluations and provides an important additional test of model generalizability that more closely simulates real-world clinical applications, in which data is generated separately from the data used to construct models. More generally, whereas MAQC-II evaluated multiple prediction problems in numerous datasets with gene expression data and samples numbers from 70 to 340, our study went deeper into a evaluating a single prediction problem, utilizing copy number and clinical information in addition to gene expression, and with a dataset of 2,000 samples in addition to an independently-generated dataset with 102 samples.

The model achieving top performance in both the initial evaluation phase and the evaluation in additional datasets combined a state-of-the-art machine learning approach (random survival forest) with a clinically motivated feature selection strategy that used all clinical features together with an aggregate genomic instability index. Interestingly, this specific model was not tested in the uncontrolled phase, and was the result of the attempt to isolate and combine aspects of different modeling approaches in a controlled experiment. The genomic instability index measure may serve as a proxy for the degree to which DNA damage repair pathways (including, for instance, housekeeping genes like p53 and RB) have become dysregulated [37].

Beyond the specifics of the top performing models, we believe the more significant contribution of this work is as a building block, providing a set of baseline findings, computational infrastructure, and proposed research methodologies used to assess breast cancer prognosis models, and extending in the future to additional phenotype prediction problems. Towards this end, we have recently extended this work into an open collaborative competition through which any researcher can freely register and evaluate the performance of submitted models against all others submitted throughout the competition. Though this expanded breast cancer competition, and future phenotype prediction competitions to be hosted as extensions of the current work, we invite researchers to improve, refute, and extend our findings and research methodologies to accelerate the long arc of cumulative progress made by the community through a more transparent and objectively assessed process.

## Methods

### Breast Cancer Prognosis Competition Design and Software

Our competition was designed to assess the accuracy of predicting patient survival (using the overall survival metric, median 10 year follow-up) based on **feature data** measured in the METABRIC cohort of 980 patients, including gene expression and copy number profiles and 16 clinical covariates (Table 1). Participants were given a **training dataset** consisting of data from 500 samples, and data from the remaining 480 were hidden from participants and used as a **validation dataset** to evaluate submitted models.

**Figure 5:**
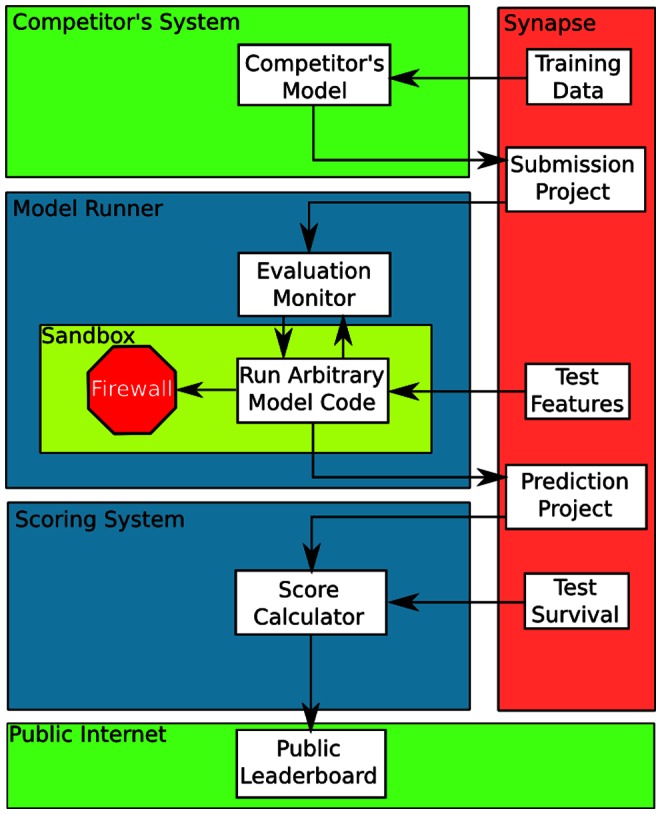
Model evaluation pipeline schematic. Green regions: Public areas, untrusted. Blue regions: Trusted areas where no competitor's code is to be run. Yellow region: Sandboxed area, where untrusted code is run on a trusted system. Red region: Permissions managed by Synapse.

We developed the computational infrastructure to support the competition within the open-source Sage Synapse software platform. Detailed documentation is available on the public competition website: https://sagebionetworks.jira.com/wiki/display/BCC/Home. The system is designed to generalize to support additional community-based competitions and consists of the following components (Figure 5):

1. The ability for participants to access training data stored in the Sage Synapse software system through programmatic APIs, with initial support built for the R programming language.
2. A programmatic API for training and testing predictive models. To date, we have developed support for models developed in the R programming language conforming to a simple interface implementing methods named customTrain and customPredict. Any model conforming to this interface can be plugged-in to the competition infrastructure, trained on the training dataset, and evaluated for prediction accuracy in the validation dataset, as well as using various cross-validation statistics.
3. The ability to upload models, including re-runnable source code, in Synapse, allowing models to be shared with the community in a fully transparent, reproducible environment.
4. An automated model evaluation system for assessing the performance of submitted models and outputting the scores to a web-based real-time leaderboard. We stress this aspect of the framework, based on the findings from previous competitions that rapid feedback is critical to motivating its participants to improve their model beyond the baseline [36].
5. Communication and social networking tools, such as wikis and discussion forums (http://support.sagebase.org).

All models are available with downloadable source code using the Synapse IDs displayed in Table S1 and Table S4. An automated script continuously monitored for new submissions, which were sent to worker nodes in a computational cluster for scoring. Each worker node ran an evaluation script, which called the submitted model's customPredict method with arguments corresponding to the gene expression, copy number, and clinical covariate values in the held-out validation dataset. This function returns a vector of predicted survival times in the validation dataset, which were used to calculate the concordance index as a measure of accuracy compared to the measured survival times for the same samples. Concordance index scores were shown in a real-time leaderboard, similar to the leaderboards displaying the models scores shown in Table S1 and Table S4.

Concordance index (c-index) is the standard metric for evaluation of survival models [53]. The concordance index ranges from 0 in the case of perfect anti-correlation between the rank of predictions and the rank of actual survival time through 0.5 in the case of predictions uncorrelated with survival time to 1 in the case of exact agreement with rank of actual survival time. We implemented a method to compute the exact value of the concordance index by exhaustively sampling all pairwise combinations of samples rather than the usual method of stochastically sampling pairwise samples. This method overcomes the stochastic sampling used in standard packages for concordance index calculation and provides a deterministic, exact statistic used to compare models.

### Study timeline

Data on the original 980 samples were obtained for this study in early January, 2012. Study design and computational infrastructure were developed from then until March 14^th^, at which point participants were given access to the 500 training samples and given 1 month to develop models in the “uncontrolled experiment” phase. During this time, participants were given real-time feedback on model performance evaluated against the held-out test set of 480 samples. After this 1-month model development phase, all models were frozen and inspected by the group to conduct post-hoc model evaluation and identify modeling strategies used to design the controlled evaluation. All models in the controlled evaluation were re-trained on the 500 training samples and re-evaluated on the 480 test samples. After all evaluation was completed based on the original 980 samples, the METABRIC2 and MicMa datasets became available, and were used to perform additional evaluations of all models, which was conducted between January 2013–March 2013. For the new evaluation, all data was renormalized to the gene level, as described below, in order to allow comparison of models across datasets performed on different platforms. Models were retrained using the re-normalized data for the same 500 samples in the original training set.

### Model source code

All model source code is available in the subfolders of Synapse ID syn160764, and specific Synapse IDs for each model are listed in Table S1 and Table S4. Data stored in Synapse may be accessed using the Synapse R client (https://sagebionetworks.jira.com/wiki/display/SYNR/Home) or by clicking the download icon on the web page corresponding to each model, allowing the user to download a Zip archive containing the source files contained in the submission.

### Datasets and normalization

The METABRIC dataset used in the competition contains gene expression data from the Illumina HT 12v3 platform and copy number data derived from experiments performed on the Affymetrix SNP 6.0 platform. In the initial round of analysis, the first 980 samples data was normalized as described in [29], corresponding to the data available in the European Genome-Phenome Archive (http://www.ebi.ac.uk/ega), accession number EGAS00000000083. Copy number data was summarized to the gene level by calculating the mean value of the segmented regions overlapping a gene. Data for use in our study are available in the Synapse software system (synapse.sagebase.org) within the folder with accession number syn160764 (https://synapse.prod.sagebase.org/#Synapse:syn160764), subject to terms of use agreements described below. Data may be loaded directly in R using the Synapse R client or downloaded from the Synapse web site.

Patients treated for localized breast cancer from 1995 to 1998 at Oslo University Hospital were included in the MicMa cohort, and 123 of these had available fresh frozen tumor material [4], [28]. Gene expression data for 115 cases obtained from an Agilent whole human genome 4×44 K one color oligo array was available (GSE19783) [54]. Novel SNP-CGH data from 102 of the MicMa samples were obtained using the Illumina Human 660k Quad BeadChips according to standard protocol. Normalized LogR values summarized to gene level were made available and are accessible in Synapse (syn1588686).

All data used for the METABRIC2 and MicMa analyses are available as subfolders of Synapse ID syn1588445. For comparison of METABRIC2 and MicMa, we standardized all clinical variables, copy number, and gene expression data across both datasets. Clinical variables were filtered out that were not available in both datasets. Data on clinical variables used in this comparison are available in Synapse.

All gene expression datasets were normalized according the supervised normalization of microarrays (snm) framework and Bioconductor package [55], [56]. Following this framework we devised models for each dataset that express the raw data as functions of biological and adjustment variables. The models were built and implemented through an iterative process designed to learn the identity of important variables. Once these variables were identified we used the snm R package to remove the effects of the adjustment variables while controlling for the effects of the biological variables of interest.

SNP6.0 copy number data was also normalized using the snm framework, and summarization of probes to genes was done as follows. First, probes were mapped to genes using information obtained from the *pd.genomewidesnp.6* Bioconductor package [57]. For genes measured by two probes we define the gene-level values as an unweighted average of the probes' data. For genes measured by a single probe we define the gene-level values as the data for the corresponding probe. For those measured by more than 2 probes we devised an approach that weights probes based upon their similarity to the first eigengene. This is accomplished by taking a singular value decomposition of the probe-level data for each gene. The percent variance explained by the first eigengene is then calculated for each probe. The summarized values for each gene are then defined as the weighted mean with the weights corresponding to the percent variance explained.

For Illumina 660k data we processed the raw files using the crlmm bioconductor R package [58]. The output of this method produces copy number estimates for more than 600k probes. Next, we summarized probes to Entrez gene ids using a mapping file obtained from the Illumina web site. For genes measured by more than two probes we selected the probe with the largest variance.

### Feature selection methods

Feature selection strategies used in the controlled experiment (identified through post-hoc analysis of the uncontrolled experiment) are described briefly in Table 3. Specific genes used in each category are available within Synapse ID syn1643406 and can be downloaded as R binaries via the Synapse web client or directly loaded in R using the Synapse R client. Most feature selection strategies are sufficiently described in Table 3, and we provide additional details on 2 methods below.

The MASP (Marginal Association with Subsampling and Prior Knowledge) algorithm employs the following procedure: all genes were first scored for association with survival (using Cox regression) in chunks of 50 randomly selected gene expression samples. This process was repeated 100 times which resulted in an overall survival association score 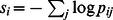 where 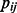 is the p-value associated with the Cox regression on the expression of gene *i* in sample set *j*. All genes were sorted in descending order by their survival association score and the top 50 oncogenes and transcription factors were kept. A list of human transcription factors was obtained from [59] and a list of oncogenes was compiled by searching for relevant keywords against the Entrez gene database.

GII is a measure of the proportion of amplified or deleted genomic loci, calculated from the copy number data. Copy number values are presented as segmented log-ratios 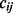 with respect to normal controls. Amplifications and deletions are thus counted when 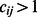 or 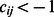
and devided by the total number of loci 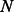.
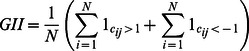



### Ethics statement

The data used in this study were collected and analyzed under approval of an IRB [29]. The MicMa study was approved by the Norwegian Regional Committee for medical research ethics, Health region II (reference number S-97103). All patients have given written consent for the use of material to research purposes.

## Supporting Information

Figure S1 Performance of models from the controlled experiment in the METABRIC2 (A) and MicMa (B) dataset.
(PNG)

Table S1 Complete details of all the models submitted to the pilot competition in the uncontrolled experimental design. Source code for all models are available using the Synapse IDs listed in this table (see Methods for description of how to view model source code).
(XLSX)

Table S2 Association of gene expression and CNA with survival and p-values of the association between gene expression and survival and between CNA and survival for the 10 probes with lowest P-value. (a) Top ten gene expression probes associated with survival marginally. (b) Top ten copy number probes associated with survival marginally. (c) Top ten gene expression probes associated with survival conditioning on clinical variables. (d) Top ten copy number alteration probes associated with survival conditioning on clinical variables.
(DOCX)

Table S3 Top 50 oncogenes and transcription factors inferred by the MASP feature selection algorithm.
(XLSX)

Table S4 Complete details of all the models evaluated in the controlled experiment. Source code for all models is available using the Synapse IDs listed in this table.
(XLSX)

Table S5 Model scores in METABRIC2 and MicMa evaluations. Models, and corresponding model scores, used in the METABRIC2 and MicMa evaluations are at syn1646909 and syn1642232, respectively.
(DOCX)
