# Supplementary figures and images for: Improving Breast Cancer Survival Analysis through Competition-Based Multidimensional Modeling
Source: PLoS Comput Biol. 2013 May 9;9(5):e1003047. doi: 10.1371/journal.pcbi.1003047 (PMC3649990; doi:10.1371/journal.pcbi.1003047)

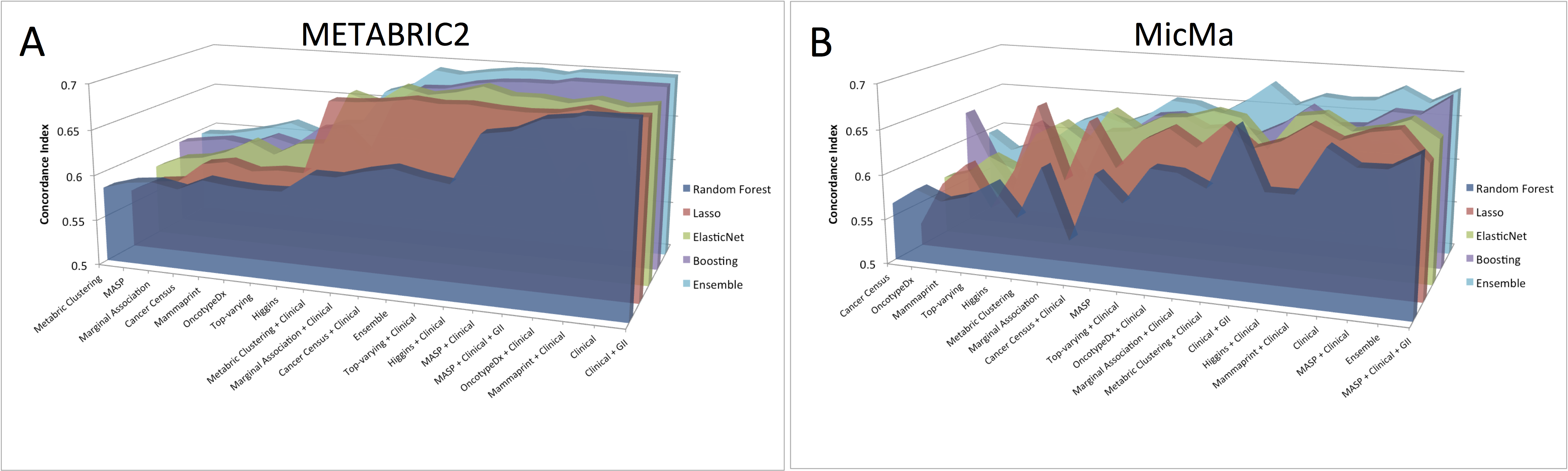

Supplement: Figure S1 — Performance of models from the controlled experiment in the METABRIC2 (A) and MicMa (B) dataset. (PNG) [file pcbi.1003047.s001.png]
