# Supplementary material for: Improving Breast Cancer Survival Analysis through Competition-Based Multidimensional Modeling
Source: PLoS Comput Biol. 2013 May 9;9(5):e1003047. doi: 10.1371/journal.pcbi.1003047 (PMC3649990; doi:10.1371/journal.pcbi.1003047)
Supplement: Table S2 — Association of gene expression and CNA with survival and p-values of the association between gene expression and survival and between CNA and survival for the 10 probes with lowest P-value. (a) Top ten gene expression probes associated with survival marginally. (b) Top ten copy number probes associated with survival marginally. (c) Top ten gene expression probes associated with survival conditioning on clinical variables. (d) Top ten copy number alteration probes associated with survival conditioning on clinical variables. (DOCX) [file pcbi.1003047.s003.docx]

| (a) |  | (b) |  | (c) |  | (d) |  |
| --- | --- | --- | --- | --- | --- | --- | --- |
| **Gene Expression Probe** | **PH p-value (marginal)** | **Copy Number Probe** | **PH p-value (marginal)** | **Gene Expression Probe** | **PH p-value (conditional)** | **Copy Number Probe** | **PH p-value (conditional)** |
| HS.579631 | 4.80E-11 | SEC24D | 5.10E-06 | LOC440160 | 1.10E-06 | PHF20 | 1.00E-05 |
| GABRE | 8.40E-09 | SPAG4L | 6.20E-06 | FLJ39632 | 1.70E-06 | DDX11 | 1.10E-05 |
| G6PD | 7.70E-08 | C20ORF185 | 6.90E-06 | HS.561314 | 2.70E-06 | IFI44L | 1.50E-05 |
| SPC24 | 1.50E-07 | C20ORF186 | 8.60E-06 | HS.579631 | 6.50E-06 | IFI44 | 1.50E-05 |
| PAK4 | 2.40E-07 | LOC149950 | 9.10E-06 | FAM13A | 7.60E-06 | HS.505141 | 1.60E-05 |
| GPI | 2.40E-07 | METAP1 | 9.90E-06 | RALGAPB | 8.00E-06 | HS.574453 | 1.60E-05 |
| MRPS5 | 2.60E-07 | BPIL3 | 1.00E-05 | LOC400455 | 1.20E-05 | SFRS11 | 3.90E-05 |
| ZIC2 | 2.60E-07 | IFI44L | 1.10E-05 | PABPC4 | 1.50E-05 | LOC149950 | 4.10E-05 |
| CBX2 | 2.70E-07 | IFI44 | 1.10E-05 | DHODH | 2.10E-05 | C20ORF152 | 4.50E-05 |
| DNMT3B | 3.40E-07 | PLXDC1 | 1.50E-05 | HS.129546 | 3.80E-05 | OVOS2 | 4.80E-05 |
