# Supplementary material for: Improving Breast Cancer Survival Analysis through Competition-Based Multidimensional Modeling
Source: PLoS Comput Biol. 2013 May 9;9(5):e1003047. doi: 10.1371/journal.pcbi.1003047 (PMC3649990; doi:10.1371/journal.pcbi.1003047)
Supplement: Table S5 — Model scores in METABRIC2 and MicMa evaluations. Models, and corresponding model scores, used in the METABRIC2 and MicMa evaluations are at syn1646909 and syn1642232, respectively. (DOCX) [file pcbi.1003047.s006.docx]

| **Gene Set** | **cIndex_MicMa** | **cIndex_METABRIC** | **Algorithms** | **Average_cIndex** |
| --- | --- | --- | --- | --- |
| Clinical + GII | 0.68008 | 0.708261803 | Random Survival Forest | 0.694170901 |
| MASP + Clinical + GII | 0.68846 | 0.69577 | Boosting | 0.692115 |
| MASP + Clinical | 0.6839899 | 0.6986667 | Ensemble | 0.6913283 |
| MASP + Clinical + GII | 0.6849679 | 0.6959604 | Ensemble | 0.69046415 |
| Mammaprint + Clinical | 0.6669461 | 0.7129527 | Ensemble | 0.6899494 |
| Clinical | 0.66583 | 0.707274046 | Random Survival Forest | 0.686552023 |
| Clinical | 0.6689019 | 0.7016373 | Ensemble | 0.6852696 |
| Mammaprint + Clinical | 0.67373 | 0.69476 | LASSO | 0.684245 |
| Higgins + Clinical | 0.66924 | 0.69905 | ElasticNet | 0.684145 |
| Mammaprint + Clinical | 0.67373 | 0.6932 | ElasticNet | 0.683465 |
| Higgins + Clinical | 0.669321 | 0.6974694 | Ensemble | 0.6833952 |
| MASP + Clinical | 0.66918 | 0.6956 | Boosting | 0.68239 |
| Higgins + Clinical | 0.66778 | 0.69405 | Boosting | 0.680915 |
| Clinical + GII | 0.6570271 | 0.7021439 | Ensemble | 0.6795855 |
| MASP + Clinical | 0.67084 | 0.68809 | LASSO | 0.679465 |
| Clinical | 0.65102 | 0.70683 | Boosting | 0.678925 |
| Mammaprint + Clinical | 0.64906 | 0.70607 | Boosting | 0.677565 |
| MASP + Clinical | 0.66281 | 0.691 | ElasticNet | 0.676905 |
| Clinical + GII | 0.64641 | 0.70687 | Boosting | 0.67664 |
| Cancer Census + Clinical | 0.65992 | 0.69271 | ElasticNet | 0.676315 |
| Metabric Clustering + Clinical | 0.66699 | 0.68411 | LASSO | 0.67555 |
| Clinical | 0.65992 | 0.6892 | LASSO | 0.67456 |
| Top-varying + Clinical | 0.65703 | 0.69095 | ElasticNet | 0.67399 |
| Cancer Census + Clinical | 0.65992 | 0.68762 | LASSO | 0.67377 |
| MASP + Clinical + GII | 0.65607 | 0.69124 | ElasticNet | 0.673655 |
| OncotypeDx + Clinical | 0.65896 | 0.68818 | LASSO | 0.67357 |
| OncotypeDx + Clinical | 0.6596 | 0.68751 | ElasticNet | 0.673555 |
| MASP + Clinical + GII | 0.66639 | 0.679642366 | Random Survival Forest | 0.673016183 |
| Higgins + Clinical | 0.65446 | 0.69084 | LASSO | 0.67265 |
| Metabric Clustering + Clinical | 0.66313 | 0.68179 | ElasticNet | 0.67246 |
| Clinical | 0.65735 | 0.68749 | ElasticNet | 0.67242 |
| Top-varying + Clinical | 0.6503213 | 0.6937443 | Ensemble | 0.6720328 |
| OncotypeDx + Clinical | 0.6402626 | 0.7030843 | Ensemble | 0.67167345 |
| Metabric Clustering + Clinical | 0.6793797 | 0.6630874 | Ensemble | 0.67123355 |
| Marginal Association + Clinical | 0.66859 | 0.67331 | ElasticNet | 0.67095 |
| Top-varying + Clinical | 0.64878 | 0.69001 | Boosting | 0.669395 |
| OncotypeDx + Clinical | 0.63481 | 0.70215 | Boosting | 0.66848 |
| Clinical + GII | 0.64451 | 0.69073 | LASSO | 0.66762 |
| Top-varying + Clinical | 0.64355 | 0.68928 | LASSO | 0.666415 |
| MASP + Clinical + GII | 0.64419 | 0.6869 | LASSO | 0.665545 |
| Marginal Association + Clinical | 0.6574462 | 0.6717148 | Ensemble | 0.6645805 |
| Marginal Association + Clinical | 0.64066 | 0.68494 | LASSO | 0.6628 |
| Cancer Census + Clinical | 0.6328583 | 0.6923174 | Ensemble | 0.66258785 |
| MASP + Clinical | 0.64823 | 0.67478 | Random Survival Forest | 0.661505 |
| Clinical + GII | 0.63102 | 0.69189 | ElasticNet | 0.661455 |
| OncotypeDx + Clinical | 0.62923 | 0.693609468 | Random Survival Forest | 0.661419734 |
| Mammaprint + Clinical | 0.61609 | 0.69735 | Random Survival Forest | 0.65672 |
| Cancer Census + Clinical | 0.62503 | 0.68145 | Boosting | 0.65324 |
| Marginal Association + Clinical | 0.63901 | 0.66215 | Boosting | 0.65058 |
| Top-varying | 0.62503 | 0.62639 | Boosting | 0.62571 |
| Higgins | 0.62699 | 0.62364 | ElasticNet | 0.625315 |
| MASP | 0.6524169 | 0.5963519 | Ensemble | 0.6243844 |
| MASP | 0.64515 | 0.60052 | Boosting | 0.622835 |
| Marginal Association + Clinical | 0.62727 | 0.6181 | Random Survival Forest | 0.622685 |
| Higgins | 0.6177703 | 0.6230905 | Ensemble | 0.6204304 |
| Higgins + Clinical | 0.61581 | 0.62176 | Random Survival Forest | 0.618785 |
| Metabric Clustering | 0.67225 | 0.56485 | LASSO | 0.61855 |
| MASP | 0.6418 | 0.59138 | ElasticNet | 0.61659 |
| Cancer Census | 0.63174 | 0.60042 | Boosting | 0.61608 |
| Metabric Clustering + Clinical | 0.63174 | 0.60042 | Boosting | 0.61608 |
| Metabric Clustering + Clinical | 0.61386 | 0.61814 | Random Survival Forest | 0.616 |
| Higgins | 0.60296 | 0.62889 | Boosting | 0.615925 |
| Marginal Association | 0.62224 | 0.60435 | Boosting | 0.613295 |
| Metabric Clustering | 0.6288069 | 0.5948046 | Ensemble | 0.61180575 |
| Marginal Association | 0.6207041 | 0.6016314 | Ensemble | 0.61116775 |
| Metabric Clustering | 0.64208 | 0.57935 | ElasticNet | 0.610715 |
| Marginal Association | 0.6228 | 0.59504 | ElasticNet | 0.60892 |
| Top-varying | 0.5956971 | 0.6204881 | Ensemble | 0.6080926 |
| Top-varying + Clinical | 0.58983 | 0.62616 | Random Survival Forest | 0.607995 |
| Mammaprint | 0.59598 | 0.61868 | ElasticNet | 0.60733 |
| Marginal Association | 0.62001 | 0.59402 | Random Survival Forest | 0.607015 |
| MASP | 0.61833 | 0.59083901 | Random Survival Forest | 0.604584505 |
| Mammaprint | 0.59933 | 0.60812 | LASSO | 0.603725 |
| Top-varying | 0.5834 | 0.62103 | ElasticNet | 0.602215 |
| Cancer Census | 0.5955574 | 0.6084145 | Ensemble | 0.60198595 |
| Mammaprint | 0.5861973 | 0.6169344 | Ensemble | 0.60156585 |
| Higgins | 0.59877 | 0.60354 | LASSO | 0.601155 |
| Mammaprint | 0.598 | 0.602 | Mammaprint | 0.6 |
| Higgins | 0.59681 | 0.59756 | Random Survival Forest | 0.597185 |
| MASP | 0.61106 | 0.57821 | LASSO | 0.594635 |
| OncotypeDx | 0.5828 | 0.6064 | OncotypeDx | 0.5946 |
| OncotypeDx | 0.5758592 | 0.6076582 | Ensemble | 0.5917587 |
| OncotypeDx | 0.57698 | 0.60493 | Boosting | 0.590955 |
| OncotypeDx | 0.58284 | 0.598259944 | Random Survival Forest | 0.590549972 |
| OncotypeDx | 0.5739 | 0.60613 | ElasticNet | 0.590015 |
| Top-varying | 0.57977 | 0.59666 | Random Survival Forest | 0.588215 |
| OncotypeDx | 0.57474 | 0.60029 | LASSO | 0.587515 |
| Marginal Association | 0.59206 | 0.58142 | LASSO | 0.58674 |
| Mammaprint | 0.57195 | 0.60152 | Random Survival Forest | 0.586735 |
| Cancer Census + Clinical | 0.54373 | 0.62651 | Random Survival Forest | 0.58512 |
| Cancer Census | 0.5658 | 0.604 | ElasticNet | 0.5849 |
| Top-varying | 0.55239 | 0.60394 | LASSO | 0.578165 |
| Metabric Clustering | 0.55937 | 0.59613 | Boosting | 0.57775 |
| Cancer Census | 0.5644 | 0.58891 | Random Survival Forest | 0.576655 |
| Mammaprint | 0.54121 | 0.61187 | Boosting | 0.57654 |
| Metabric Clustering | 0.56245 | 0.58257 | Random Survival Forest | 0.57251 |
| Cancer Census | 0.52501 | 0.60338 | LASSO | 0.564195 |
